# An increase in glucosylceramide synthase induces Bcl-xL-mediated cell survival in vinorelbine-resistant lung adenocarcinoma cells

**DOI:** 10.18632/oncotarget.4109

**Published:** 2015-05-12

**Authors:** Wei-Hsin Chiu, Wu-Chou Su, Chia-Ling Li, Chia-Ling Chen, Chiou-Feng Lin

**Affiliations:** ^1^ Division of Hemato-Oncology, Department of Internal Medicine, National Cheng Kung University Hospital, Tainan, Taiwan; ^2^ Center for Translational Medicine, Taipei Medical University, Taipei, Taiwan; ^3^ Graduate Institute of Medical Sciences, College of Medicine, Taipei Medical University, Taipei, Taiwan; ^4^ Department of Microbiology and Immunology, College of Medicine, Taipei Medical University, Taipei, Taiwan

**Keywords:** glucosylceramide synthase, vinorelbine, Bcl-xL, lung cancer, multiple drug-resistances

## Abstract

Reversing drug resistance with concurrent treatment confers anticancer benefits. In this study, we investigated the potential mechanism of glucosylceramide synthase (GCS)-mediated vinca alkaloid vinorelbine (VNR) resistance in human lung adenocarcinoma cells. Compared with PC14PE6/AS2 (AS2) and CL1-0 cells, apoptotic analysis showed that both A549 and CL1-5 cells were VNR-resistant, while these cells highly expressed GCS at the protein level. VNR treatment significantly converts ceramide to glucosylceramide in VNR-resistant cells; however, pharmacologically inhibiting GCS with (±)-*threo*-1-Phenyl-2-decanoylamino-3-morpholino-1-propanol hydrochloride (PDMP) induced ceramide accumulation, accompanied by a decrease in glucosylceramide. Under concurrent treatment with VNR and PDMP, an increase in cell apoptosis could be identified; furthermore, genetically silencing GCS confirmed these effects. In VNR-resistant cells, Bcl-xL expression was aberrantly increased, while pharmacologically inhibiting Bcl-xL with ABT-737 sensitized cells to VNR-induced apoptosis. Conversely, enforced expression of Bcl-xL strengthened the survival response of the VNR-susceptible cells AS2 and CL1-0. Without changes in mRNA expression, Bcl-xL was overexpressed independent of β-catenin-mediated transcriptional regulation in VNR-resistant cells. Simultaneous GCS inhibition and VNR treatment caused a decrease in Bcl-xL expression. According to these findings, an increase in GCS caused Bcl-xL augmentation, facilitating VNR resistance in lung adenocarcinoma cells.

## INTRODUCTION

Lung cancer currently remains a major cause of cancer deaths worldwide. The prognosis of lung cancer is poor, and resistance to chemotherapy is the greatest obstacle to effective cancer treatment. There are numerous mechanisms related to drug resistance in lung cancers, including ineffective drug delivery to the tumors, increased drug efflux, DNA repair, drug inactivation, the interference of target enzymes, the shortened half-lives of drugs, apoptosis defects, lack of drug specificity to the tumors, and tumor vasculature [1-7]. Cells generate ceramide in response to stresses such as chemotherapy, causing proliferation arrest, apoptosis, or autophagy [8]. However, cancer cells eliminate ceramide through ceramide glycosylation to escape death, and persistently promoting ceramide glycosylation can select cancer cells for drug resistance [9-12].

Multiple drug-resistant (MDR) cancers have elevated glucosylceramide synthase (GCS) and P-glycoprotein, and GCS promoter activity is 15-fold higher in MCF-7-AdrR cells than in MCF-7 cells [13]. Attenuating GCS expression and/or activity with inhibitors or oligonucleotides selectively reverses drug resistance in cancer cells [14, 15]. GCS up-regulates *MDR1* mRNA expression for cancer drug resistance through c-Src and β-catenin [16]. Leukemia cells with GCS overexpression also show increased levels of MDR1 and Bcl-2 expression, as well as a poor response to chemotherapy [17]. Vinorelbine (VNR) is initially developed in 1979, and is a semi-synthetic second generation vinca-alkaloid [18]. VNR binds to tubulin as a potent inhibitor of mitotic microtubule polymerization in chemotherapy, and causes aberrant ROS-mediated JNK activation, Mcl-1 downregulation, DNA damage, mitochondrial dysfunction, and apoptosis in lung adenocarcinoma cells [25]. Further phase III studies demonstrate the application of vinorelbine in excellent combination with platinum in lung cancer patients [19-21]. It has been used both as a single agent and in combination with cisplatin for first-line treatment of advanced and metastatic non-small cell lung cancer [19, 22-24]; however, tumors also develop resistance in response to VNR treatment. The possible relationship between VNR resistance and GCS expression has not been explored.

The Bcl-2 family proteins, including pro-apoptotic proteins (Bax, BAD, Bak, BIM, BID, …etc.) and anti-apoptotic proteins (including Bcl-2, Bcl-xL, Mcl-1, …etc.), control mitochondrial outer membrane permeabilization [25]. Bcl-2 down-regulation was found to be a mechanism of paclitaxel resistance [26]. Expression of Bcl-xL in several cancer cells could induce MDR [27]. In gastric cancers, MDR-1 behaves as an oncofetal protein and had anti-apoptotic action through cross-talk with Bcl-xL [28]. Basically, MDR-1, Bcl-xL, *H. pylori*, and Wnt/β-catenin signaling contribute to gastric carcinogenesis [29]. β-catenin-transduced regulatory T cells showed decreases in c-myc and Bax but an increase in Bcl-xL [30]. In this study, we further examined a possible mechanism by which high expression of GCS induced Bcl-xL-mediated anti-apoptosis in VNR-resistant lung cancer cells.

## RESULTS

### High GCS is expressed in lung cancer cells resistant to VNR

**Figure 1:**
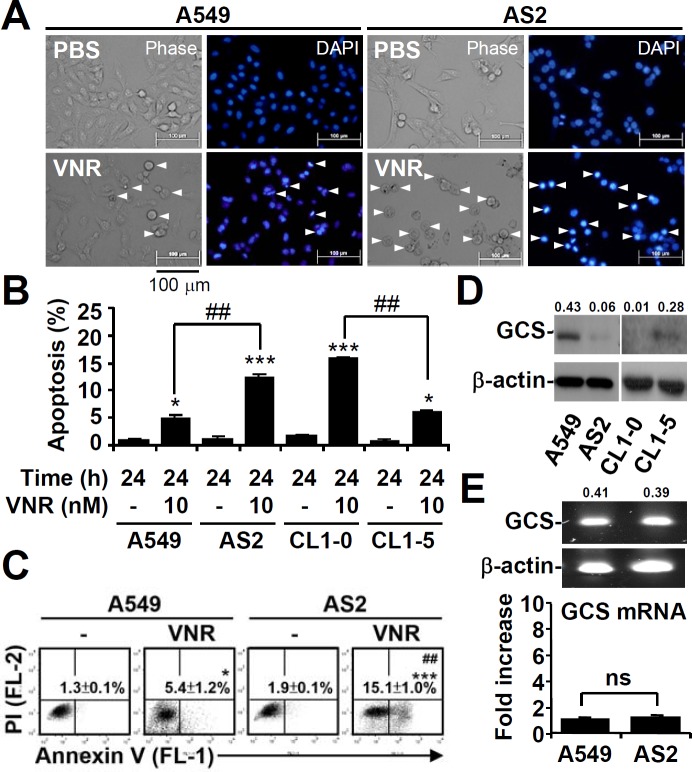
High expression of GCS in lung cancer cells resistant to VNR-induced apoptosis **A.** A549 and AS2 cells were treated with VNR (10 nM) for 24 h. Representative images of apoptotic (DNA condensation, arrowheads) cells stained with DAPI, followed by fluorescence microscopic observation. **B.** Nuclear PI staining and subsequent flow cytometric analysis determined cell apoptosis in VNR-treated A549, AS2, CL1-0, and CL1-5 cells. The percentages (%) of apoptotic cells are shown as the means ± SDs of three individual experiments. *P < 0.05 and ***P < 0.001 compared with untreated controls. ##*P* < 0.01. **C.** Annexin V/PI staining and subsequent flow cytometric analysis determined cell apoptosis in VNR-treated A549 and AS2 cells. The percentages (%) of apoptotic cells (annexin V^+^ PI^−^) are shown as the means ± SDs of three individual experiments. *P < 0.05 and ***P < 0.001 compared with untreated controls. ##*P* < 0.01. **D.** Representative western blot analysis showing the expression of GCS in A549, AS2, CL1-0, and CL1-5 cells. β-actin was used as an internal control. The relative ratios of the measured proteins with those for β-actin are also shown. **E.** RT-PCR assay showing the mRNA expression of GCS in A549 and AS2 cells. The relative densities of the measured mRNA with those for β-actin are also shown. The data, compared with the normalized values of A549 cells, are shown as the means ± SDs of three individual experiments. ns, not significant.

VNR works as an anticancer agent by inducing cell growth inhibition and cell apoptosis. In our previous study, A549 cells were much less susceptible to VNR-induced apoptosis than AS2 cells [31]. We examined the cytotoxic effects of VNR using fluorescence microscopy. These analyses showed that VNR treatment caused shrinkage of A549 and AS2 cells (Figure 1A), and DAPI staining confirmed the presence of apoptotic cells with DNA condensation in VNR-treated cells. Nuclear PI staining (Figure 1B) and annexin V/PI staining (Figure 1C), followed by flow cytometry, all revealed that VNR significantly (*P* < 0.05) induced more apoptosis in AS2 and CL1-0 cells than in A549 and CL1-5 cells. Western blot analysis showed that A549 and CL1-5 cells had higher GCS expression than AS2 and CL1-0 cells (Figure 1D). However, RT-PCR assays showed that there was no difference in the mRNA expression of GCS in AS2 and A549 cells (Figure 1E). These results demonstrated that high GCS expression in lung cancer cells resistant to VNR and GCS expression was not regulated by mRNA transcription.

### Blockage of GCS induces ceramide accumulation with decreased glucosylceramide

**Figure 2:**
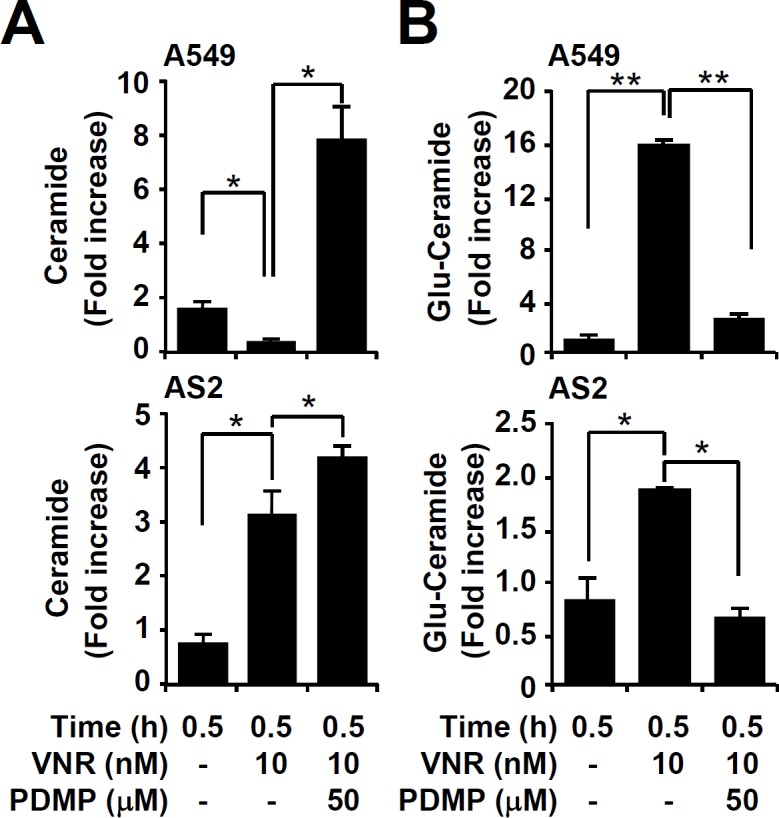
Pharmacologically inhibiting GCS induces ceramide accumulation in VNR-treated A549 and AS2 cells Immunostaining followed by flow cytometric analysis, showing the levels of ceramide **A.** and glucosylceramide (Glu-Ceramide) **B.** in A549 and AS2 cells treated with VNR without and with PDMP. The mean fluorescence intensity of each stain, compared with the normalized values of untreated cells (fold increase), is shown as the means ± SDs of three individual experiments. *P < 0.05 and **P < 0.01.

Ceramide immunostaining, followed by flow cytometry, showed that VNR treatment caused a significant increase in AS2 but not A549 cells. Inhibiting GCS with PDMP all significantly (*P* < 0.05) induced ceramide expression in A549 and AS2 cells, compared to VNR treatment only (Figure 2A). We also investigated the levels of glucosylceramide because the sphingolipid metabolites are typically regulated during ceramide expression. Ceramide levels are tightly regulated through different pathways including *de novo* synthesis, hydrolysis of sphingomyelin, and decreasing ceramide metabolism. In the metabolic pathway, ceramide converts to glucosylceramide, sphingosine-1-phosphate, and ceramide-1-phosphate by glucosylceramide synthase, ceramidase, and ceramide kinase, respectively [8, 32]. A significant increased generation of glucosylceramide was found in VNR-treated A549 cells, as compared to AS2 cells. Furthermore, PDMP decreased glucosylceramide generation in VNR-treated A549 and AS2 cells, compared to VNR treatment alone (*P* < 0.05) (Figure 2B). These results demonstrate that inhibiting GCS caused ceramide generation, followed by decreased glucosylceramide.

### Inhibition of GCS causes significant apoptosis in high GCS expressing cancer cells

**Figure 3:**
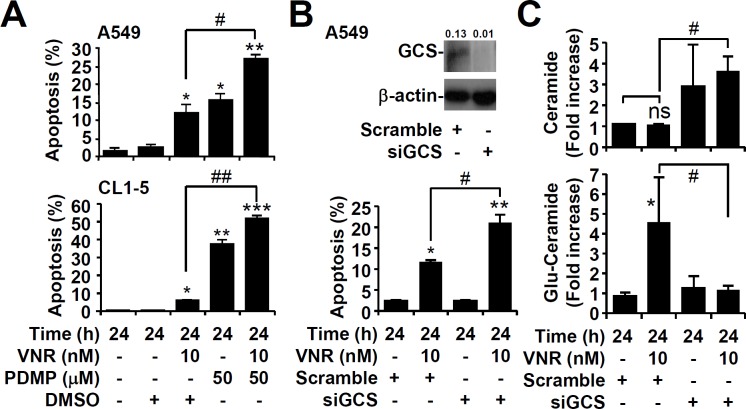
Inhibition of GCS causes significant apoptosis in high GCS expressing cancer cells **A.** Following VNR stimulation in PDMP-treated A549 and CL1-5 cells, nuclear PI staining and subsequent flow cytometric analysis determined cell apoptosis, and the percentages (%) of apoptotic cells are shown as the means ± SDs of three individual experiments. DMSO was used as a control. *P < 0.05, **P < 0.01, and ***P < 0.001, compared with untreated controls. #*P* < 0.05 and ##*P* < 0.01. **B.** Representative western blotting showing the expression of GCS in scramble- and siGCS-transfected A549 cells. β-actin was used as an internal control. The relative ratios of the measured proteins with those for β-actin are also shown. Following VNR stimulation and PI-based flow cytometric analysis, the percentages (%) of apoptotic cells are shown as the means ± SDs of three individual experiments. *P < 0.05 and **P < 0.01, compared with untreated controls. #*P* < 0.05. **C.** Immunostaining followed by flow cytometric analysis, showing the levels of ceramide and glucosylceramide (Glu-Ceramide) in scramble- and siGCS-transfected A549 cells treated with VNR. The mean fluorescence intensity of each stain, compared with the normalized values of untreated cells (fold increase), is shown as the means ± SDs of three individual experiments. *P < 0.05, compared with untreated control. #*P* < 0.05. ns, not significant.

Because A549 and CL1-5 cells were resistant to VNR, we next examined the role of GCS in our model. Blocking GCS plus VNR facilitated more apoptosis than VNR alone in A549 and CL1-5 cells (*P* < 0.01) (Figure 3A). We knocked down GCS with siRNA (Figure 3B, upper), and VNR plus GCS knockdown induced more apoptosis than VNR alone in A549 cells (*P* < 0.05) (Figure 3B, lower). The generation of ceramide (Figure 3C, upper) and glucosylceramide (Figure 3C, lower) in VNR-treated A549 cells with or without GCS knockdown were confirmed as similar to the results of PDMP treatment. These results demonstrated that GCS played an important role in the VNR resistance mechanism.

### Overexpression of Bcl-xL results in resistance to VNR

**Figure 4:**
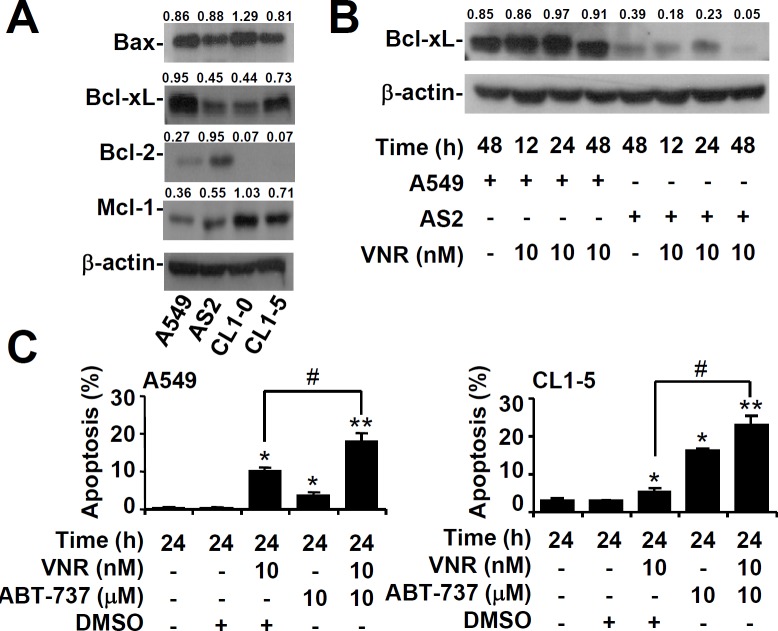
Inhibiting Bcl-xL facilitates VNR-induced apoptosis in VNR-resistant cells Representative western blot analysis showing the expression of Bax, Bcl-xL, Bcl-2, and Mcl-1 in A549, AS2, CL1-0, and CL1-5 cells **A.** and the expression of Bcl-xL in VNR-treated A549 and AS2 cells **B.**. β-actin was used as an internal control. The relative ratios of the measured proteins with those for β-actin are also shown. **C.** In the presence of the Bcl-xL inhibitor ABT-737, A549 and CL1-5 cells were treated with VNR. Nuclear PI staining and subsequent flow cytometric analysis determined apoptosis, and the percentages (%) of apoptotic cells are shown as the means ± SDs of three individual experiments. DMSO was used as a control. *P < 0.05 and **P < 0.01, compared with untreated controls. #*P* < 0.05.

**Figure 5:**
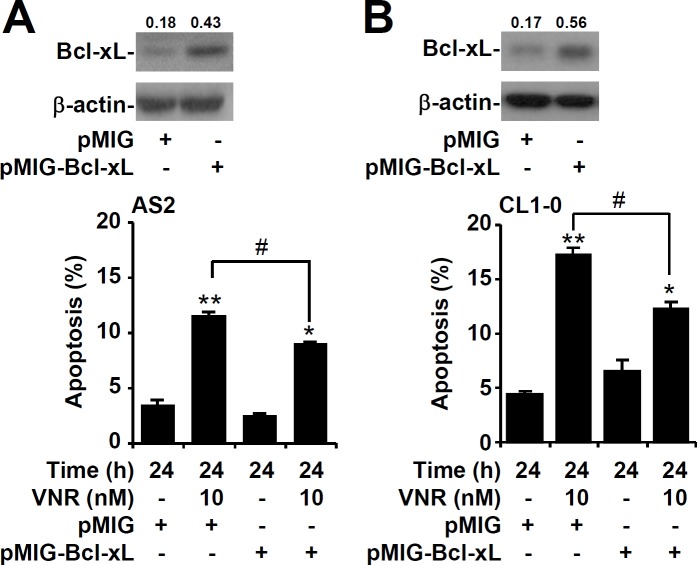
Overexpression of Bcl-xL in AS2 and CL1-0 cells resistant to VNR-induced apoptosis Representative western blotting showing the expression of Bcl-xL in AS2 **A.** and CL1-0 **B.** cells without or with the transfection of plasmids containing pMIG-Bcl-xL. pMIG was used as a vector control. β-actin was used as an internal control. The relative ratios of the measured proteins with those for β-actin are also shown. Following VNR stimulation, nuclear PI staining and subsequent flow cytometric analysis determined apoptosis, and the percentages (%) of apoptotic cells are shown as the means ± SDs of three individual experiments. *P < 0.05 and **P < 0.01, compared with untreated controls. #*P* < 0.05.

The Bcl-2 family of proteins includes both pro- and anti-apoptotic molecules, so we next examined the expression of these proteins in lung cancer cells. Western blot analysis showed that expression of Bcl-xL was lower in AS2 and CL1-0 lung cancer cells than in A549 and CL1-5 cells (Figure 4A). The Bcl-xL level in AS2 and A549 lung cancer cells decreased gradually 48 h after VNR was treated (Figure 4B). Nuclear PI staining, followed by flow cytometry, revealed that inhibiting Bcl-xL with Abt737 plus VNR contributed to more apoptosis in A549 and CL1-5 lung cancer cells than VNR alone (*P* < 0.05) (Figure 4C). Overexpression of Bcl-xL resulted in more resistance to VNR in AS2 (*P* < 0.05) (Figure 5A) and CL1-0 lung cancer cells (*P* < 0.05) (Figure 5B). These results demonstrated that expression of Bcl-xL played an important role in modulating the response of VNR.

### β-catenin increased in A549 cells independent of Bcl-xL

**Figure 6:**
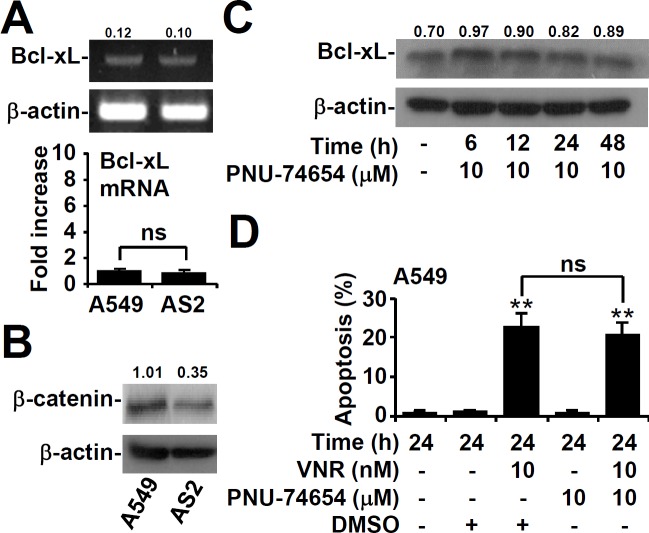
β-catenin is not required for Bcl-xL expression or VNR-induced apoptosis in A549 cells **A.** RT-PCR assay showing the mRNA expression of Bcl-xL in A549 and AS2 cells. The relative densities of the measured mRNA with those for β-actin are also shown. The data, compared with the normalized values of A549 cells, are shown as the means ± SDs of three individual experiments. ns, not significant. Representative western blot analysis showing the expression of β-catenin in A549 and AS2 cells **B.** and of Bcl-xL in β-catenin inhibitor PNU-74654-treated A549 cells **C.**. β-actin was used as an internal control. The relative ratios of the measured proteins with those for β-actin are also shown. **D.** At the same time, nuclear PI staining and subsequent flow cytometric analysis determined apoptosis, and the percentages (%) of apoptotic cells are shown as the means ± SDs of three individual experiments. **P < 0.01, compared with the relative controls. ns, not significant.

RT-PCR assays showed that there was no difference in the mRNA expression of Bcl-xL between AS2 and A549 cells (Figure 6A). Previous publications have shown that β-catenin enhanced the expression of Bcl-xL [30], so we next examined the relationship between these molecules. There was higher expression of β-catenin in A549 cells than in AS2 cells by western blot analysis (Figure 6B). Pharmacologically inhibiting β-catenin with PNU74654 did not affect the Bcl-xL level (Figure 6C) or apoptosis (Figure 6D). These results demonstrated that β-catenin was not essential for Bcl-xL to increase in A549 cells.

### Inhibiting GCS decreases the expression of Bcl-xL

**Figure 7:**
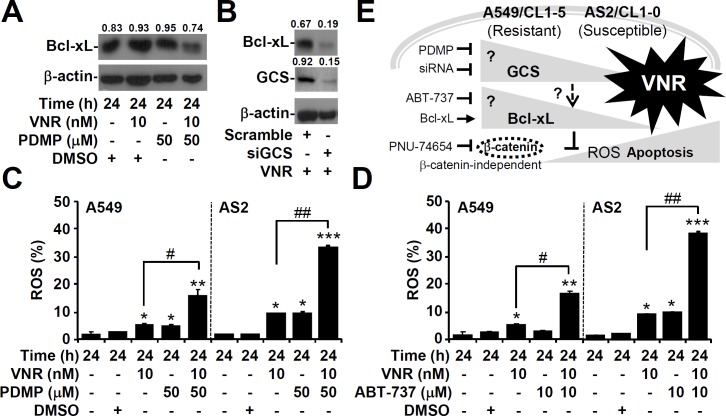
Inhibiting GCS decreases Bcl-xL expression in VNR-treated A549 cells Representative western blot analysis showing the expression of Bcl-xL in VNR-treated A549 cells **A.** and in VNR-treated A549 cells with siGCS transfection **B.**. DMSO and scramble were used as the controls. β-actin was used as an internal control. The relative ratios of the measured proteins with those for β-actin are also shown. CM-H_2_DCFDA staining, followed by flow cytometric analysis, was used to determine the levels of ROS in VNR-treated A549 and AS2 cells for 24 h with or without PDMP **C.** and ABT-737 **D.** co-treatment. DMSO was used as a negative control. For flow cytometric analyses, the percentages are the means ± SDs of three individual experiments. *P < 0.05, **P < 0.01, and ***P < 0.001, compared with untreated controls. #*P* < 0.05 and ##*P* < 0.01. **E.** A hypothetic model of this work. Targeting GCS and Bcl-xL confers benefits for facilitating VNR-induced cell apoptosis in lung adenocarcinoma, particularly in VNR-resistant cells.

To explore the relationship between GCS and Bcl-xL, blocking GCS with PDMP caused decreased Bcl-xL levels in A549 cells (Figure 7A), and GCS knockdown also diminished the expression of Bcl-xL (Figure 7B). Furthermore, CM-H_2_DCFDA staining, followed by flow cytometric analysis, demonstrated that VNR plus PDMP (Figure 7C) or ABT-737 (Figure 7D) significantly increased the generation of intracellular ROS, both in A549 (*P* < 0.05) and in AS2 (*P* < 0.01) cells. These results demonstrated that GCS could modulate Bcl-xL expression.

## DISCUSSION

As summarized in Figure 7E, this study demonstrated a VNR-resistant strategy in lung adenocarcinoma cells, by which an increase GCS expression caused anti-apoptotic Bcl-xL up-regulation to facilitate cancer cell survival in response to VNR treatment. While this study explored why lung cancer cells overexpressed GCS, it remains unclear how cancer cells acquire GCS overexpression. Without changes in the level of mRNA, a post-modification for GCS up-regulation is speculated. Furthermore, in a future study, an *in vivo* model is needed to confirm the combined chemotherapeutic effects of VNR and GCS inhibition on GCS-overexpressing lung cancers.

GCS overexpression was found in breast cancers with metastasis but not in benign fibroadenomas or primary tumors [33]. We theorized that different GCS-expressing lung cancer would have variable responses to chemotherapy. Recently, more publications have reported MDR cancers having increased GCS mRNA, protein, and P-glycoprotein [13]. In the future, inhibiting GCS could be an important target for patients with VNR-resistant lung cancer. Gaucher disease type 1 would be expected to be treated with Cerdelga (eliglustat) in the future. Cerdelga is an orally administered GCS inhibitor that reduces the production of glucosylceramide. The symptoms of Gaucher disease are caused by the accumulation of glucosylceramide in cells and tissues, leading to abnormal blood counts, an enlarged liver or spleen, and destructive bone disease. A new drug application filing was based on the data from two completed phase III trials: ENGAGE and ENCORE [34]. We expect to observe cytotoxic effects with new GCS inhibitors, facilitating the use of different chemotherapeutic agents in drug-resistant cancer cell lines. Our results also demonstrated that PDMP and GCS siRNA induced different levels of cell apoptosis. It was surmised that PDMP may induce off-target effects and the selection of cells with GCS silence may cause cells gain of survival. For patients with MDR cancers, chemotherapeutic agents combined with drugs inhibiting GCS should be investigated in our future work.

Our previous study demonstrated that concurrent chemoradiotherapy induced an increase in ceramide, accompanied by a decrease in glucosylceramide that was positively correlated with the cytotoxic effects [24]. In A549 cells with high GCS expression, VNR caused low ceramide accumulation, following by elevated glucosylceramide. Thus, inhibiting GCS induced more cell death in high GCS-expressing lung cancer cells, and the major cytotoxic effects were caused by ceramide accumulation. However, no publications thus far have investigated how high GCS-expressing cancer cells resisted chemotherapy-induced apoptosis. To our knowledge, our study was the first research to explore the relationships among GCS, Bcl-xL, and VNR susceptibility. We analyzed the expression of the anti-apoptotic proteins Bcl-2, Bcl-xL, and Mcl-1 in different lung cancer cells, and high GCS-expressing cancer cells also showed markedly increased Bcl-xL expression. VNR was treated using A549 and AS2 cells, but Bcl-xL expression did not show a dominant change until GCS was inhibited.

Inhibiting Bcl-xL induced more apoptosis in high GCS-expressing cancer cells. We hypothesized that Bcl-xL would be the key point in determining VNR resistance in high GCS-expressing cancer cells. Overexpression of Bcl-xL was designed to prove its anti-apoptotic role in low GCS-expressing cancer cells. Our results demonstrated that overexpressed Bcl-xL could induce a poor response to VNR. GCS caused VNR resistance through the anti-apoptotic effects of Bcl-xL. However, lung cancer cells had different Bcl-xL levels but not due to transcription. It was surmised that a less Bcl-xL in CL1-5 cells may increase the cellular sensitivity to ABT-737 treatment. Bcl-xL post-transcriptional modification or degradation might affect VNR resistance. Keap1 degrades Bcl-xL via phosphoglycerate mutase 5 [35], but it was not involved in our model (data not shown). Post-modification of Bcl-xL for its protein stability remains to be investigated in VNR-sensitive and -insensitive lung cancer cells.

Because GCS up-regulates *MDR1* expression for cancer drug resistance through *cSrc* and β-catenin [16], we hypothesized that Src and β-catenin might be the target points. In addition, Ding et al. found that β-catenin-transduced Treg cells showed enhanced Bcl-xL expression [30]. We designed this study to explore the relationships among Src, β-catenin, and Bcl-xL. However, inhibiting β-catenin did not reduce the expression of Bcl-xL, indicating an independent role for β-catenin in the Bcl-xL increase in our model. However, inhibiting Src plus VNR could result in more apoptosis than VNR alone (Figure S1). We believe that Src plays some role in VNR resistance. Blocking GCS through inhibition or knockdown of PDMP could result in decreased Bcl-xL expression, demonstrating that GCS contributed to Bcl-xL-mediated cell survival in VNR-resistant lung cancer cells.

## MATERIALS AND METHODS

### Cell culture and reagents

The human lung adenocarcinoma PC14PE6/AS2 (AS2) cell line was established from ascites generated from PC14PE6 cells (a gift from Isaiah J. Fidler; MD Anderson Cancer Center, Houston, TX, USA) in nude mice and CL1-0 and CL1-5 cells were kindly provided by Dr. Pan-Chyr Yang (Department of Internal Medicine, National Taiwan University Hospital). AS2 and human lung adenocarcinoma A549 (CCL185, ATCC), CL1-0, and CL1-5 cells were routinely grown on plastic in Dulbecco's modified Eagle's medium (Gibco-BRL; Grand Island, NY, USA) with L-glutamine and 15 mM HEPES, supplemented with 10% fetal bovine serum (Gibco-BRL), 100 units of penicillin, and 100 μg/ml streptomycin and maintained at 37°C in 5% CO_2_. Other chemical drugs used for cell culture were purchased from Sigma-Aldrich (St. Louis, MO, USA). The vinca alkaloids VNR, Bcl-xL inhibitor ABT-737, β-catenin inhibitor PNU-74654 was purchased from Sigma-Aldrich. The GCS inhibitor D,L-*threo*-1-phenyl-2-decanoylamino-3-morpholino-1-propanol hydrochloride (PDMP) was obtained from Sigma-Aldrich and dissolved in dimethyl sulfoxide (DMSO) or ethanol prior to dilution with PBS. Rabbit anti-human Bax, Bcl-xL, Bcl-2, Mcl-1, and β-catenin were purchased from Cell Signaling Technology (Beverly, MA, USA). Antibodies against GCS, ceramide, and glucosylceramide (Glc-Ceramide) were obtained from Sigma-Aldrich. β-actin antibodies and horseradish peroxidase-conjugated or Alexa 488-conjugated anti-rabbit IgG were obtained from Chemicon International (Temecula, CA, USA).

### Cell apoptosis assays

To observe nuclear condensation, 4′,6-diamidino-2-phenylindole (DAPI; Sigma-Aldrich)-stained cells were observed using a fluorescence microscope (IX71; Olympus, Tokyo, Japan). Cell apoptosis levels were analyzed using nuclear propidium iodide (PI; Sigma-Aldrich) staining and flow cytometry (FACSCalibur; Becton Dickinson, San Jose, CA) with the excitation set at 488 nm and emission detected with the FL-2 channel (565-610 nm). The distribution of cells in the different phases of the cell cycle was calculated using MetaMorph software (Molecular Devices, Downingtown, PA, USA). Annexin V/PI staining was performed according to the manufacturer's instructions (eBioscience, San Diego, CA, USA). The cells were detected in the FL-1 (480–530 nm) and FL-2 channels (565–610 nm) using the FACS Calibur. For apoptosis analysis, the samples were analyzed using CellQuest Pro 4.0.2 software (Becton Dickinson), and quantification was performed using WinMDI 2.8 software (The Scripps Institute, La Jolla, CA, USA). Apoptosis levels are reported as the percentage (%) of cells in the sub-G_1_ phase and in the gate of annexin V^+^ PI^−^ cells.

### Western blot analysis

Harvested cells were lysed with a buffer containing 1% Triton X-100, 50 mM of Tris (pH 7.5), 10 mM of EDTA, 0.02% NaN_3_, and a protease inhibitor cocktail (Roche Boehringer Mannheim Diagnostics, Mannheim, Germany). Following one cycle of freeze-thaw, cell lysates were centrifuged at 10,000 × *g* at 4°C for 20 min. Lysates were boiled in sample buffer for 5 min. The proteins were then subjected to SDS-PAGE and transferred to PVDF membrane (Millipore, Billerica, MA, USA) using a semi-dry electroblotting system. After blocking with 5% skim milk in PBS, the membranes were incubated with a 1/1000 dilution of primary antibodies at 4°C overnight. The membranes were then washed with 0.05% PBS-Tween 20 and incubated with a 1/5000 dilution of horseradish peroxidase-conjugated secondary antibodies at room temperature for 1 h. After washing, the membranes were soaked in ECL solution (PerkinElmer Life Sciences Inc., Boston, MA) for 1 min, and then exposed to film (BioMax; Eastman Kodak, Rochester, NY, USA). The relative optical density (OD) of signal protein was quantified using ImageJ software (version 1.41o) from W. Rasband (National Institutes of Health, Bethesda, MD, USA).

### RT-PCR

Total cellular RNA was extracted using an Ultraspec-II RNA isolation system (Biotecx, Houston, TX, USA), following the manufacturer's instructions. The concentration of RNA was quantified by spectrophotometry at 260 nm (U-2000; Hitachi, Tokyo, Japan). The cDNA, in a total volume of 100 μl, was prepared after reverse transcription of cellular RNA (20 μg) with Moloney murine leukemia virus reverse transcriptase (Promega, Madison, WI, USA) using an 18-mer oligo(dT) as the primer. The cDNA (3 μl) was added to the PCR buffer containing primers (1.5 μM each), MgCl_2_ (1.5 mM), dNTPs (0.2 mM each), and *Taq* DNA polymerase (1 U; Promega) in a total reaction volume of 50 μl. The oligonucleotide primers for human GCS (5′-GACCTGGCCTTGGAGGGAAT-3′ and 5′-GAGACACCTGGGAGCTTGCT-3′), Bcl-xL (5′-GCTGGGACACTTTTGTGGAT-3′ and 5′-TGTCTGGTCACTTCCGACTG-3′), and β-actin (5′-CTCCTTAATGTCACGCACGAT-3′ and 5′-CATGTACGTTGCTATCCAGGC-3′) were used. Thirty cycles were performed (95°C for 1 min, 55°C for 2 min, and 72°C for 3 min) using a PCR controller (GeneAmp PCR System 2400; PerkinElmer, Wellesley, MA, USA). The PCR products were separated by 1.5% agarose gel electrophoresis, stained with ethidium bromide, and viewed with UV light. The relative OD of signal protein was quantified using ImageJ software.

### Immunostaining

To detect the expression of sphingolipid metabolites, we fixed, stained, and analyzed the cells. Briefly, the cells were fixed and permeabilized with 3.7 % formaldehyde in PBS. After the cells were washed and stained with primary antibody followed by an Alexa 488-conjugated secondary antibody, the cells were analyzed using flow cytometry (FACSCalibur). The samples were analyzed using CellQuest Pro 4.0.2 software, and quantification was performed using WinMDI 2.8 software. The mean fluorescence intensity of each stain, compared with the normalized values of untreated cells (fold increase), is shown.

### Transfection

GCS expression was silenced using commercialized siRNA (sc-45404) from Santa Cruz Biotechnology (Santa Cruz, CA, USA). Transfection was performed by electroporation, using a pipette-type microporator (Microporator system; Digital Bio Technology, Suwon, Republic of Korea). After transfection, the cells were incubated for 18 h in RPMI medium at 37°C before infection. A nonspecific scrambled siRNA kit (StealthTM RNAi Negative Control Duplexes, 12935-100; Invitrogen, San Diego, CA, USA) was used as the negative control. Transient transfection was performed using an MP-100 Microporator (Digital Biotechnology), according to the manufacturer's instructions for optimization and usage. The plasmid expressing pMIG-Bcl-xL and its control pMIG were purchased from Addgene (Cambridge, MA, USA) and were used in this study. After transfection, the cells were cultured for 24 h prior to the experiments.

### Intracellular ROS assay

Intracellular oxidative stress was measured by dichlorodihydrofluorescein diacetate oxidation. Cells were exposed to 20 μM 5-(and-6)-chloromethyl-2′,7′-dichlorodihydrofluorescein diacetate, acetyl ester (CM-H_2_DCFDA) (Invitrogen) for 1 h. The cells were detected with the FL-1 channel (515-545 nm) by FACSCalibur. The data was analyzed analyzed using CellQuest Pro 4.0.2 software, and quantification was done using WinMDI 2.8 software. Small cell debris was excluded by gating on a forward scatter plot.

### Statistical analysis

The values are provided as the means ± standard deviations (SDs). The groups were compared using Student's two-tailed unpaired *t* test; significance was set at a *P*-value of < 0.05.

## SUPPLEMENTARY FIGURE


