# Supplementary material for: An increase in glucosylceramide synthase induces Bcl-xL-mediated cell survival in vinorelbine-resistant lung adenocarcinoma cells
Source: Oncotarget. 2015 May 12;6(24):20513–24. doi: 10.18632/oncotarget.4109 (PMC4653022; doi:10.18632/oncotarget.4109)
Supplement: Supplementary file 1 [file oncotarget-06-20513-s001.pdf]

An increase in glucosylceramide synthase induces Bcl-xL-mediated cell survival in vinorelbine-resistant lung adenocarcinoma cells

Supplementary Material

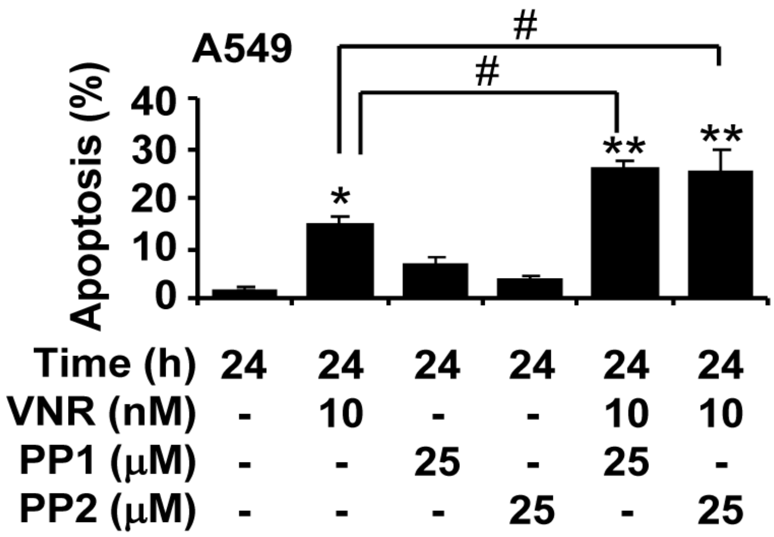

Fig. S1. Pharmacologically inhibiting Src enhances VNR-induced apoptosis. In the presence of the Src inhibitors PP1 and PP2, A549 cells were treated with VNR. Nuclear PI staining and subsequent flow cytometric analysis determined apoptosis, and the percentages (%) of apoptotic cells are shown as the means ± SDs of three individual experiments. DMSO was used as a control. *$P < 0.05$ and ***$P < 0.001$, compared with untreated controls. ###$P < 0.001$.
